# Cigarette Smoking Causes Hearing Impairment among Bangladeshi Population

**DOI:** 10.1371/journal.pone.0118960

**Published:** 2015-03-17

**Authors:** Ahmed Faisal Sumit, Anindya Das, Zinat Sharmin, Nazmul Ahsan, Nobutaka Ohgami, Masashi Kato, Anwarul Azim Akhand

**Affiliations:** 1 Department of Genetic Engineering and Biotechnology, University of Dhaka, Dhaka, Bangladesh; 2 Nutritional Health Science Research Center, Chubu University, Kasugai, Japan; 3 Department of Occupational and Environmental Health, Nagoya University Graduate School of Medicine, Nagoya, Japan; Beijing University of Chemical Technology, CHINA

## Abstract

Lifestyle including smoking, noise exposure with MP3 player and drinking alcohol are considered as risk factors for affecting hearing synergistically. However, little is known about the association of cigarette smoking with hearing impairment among subjects who carry a lifestyle without using MP3 player and drinking alcohol. We showed here the influence of smoking on hearing among Bangladeshi subjects who maintain a lifestyle devoid of using MP3 player and drinking alcohol. A total of 184 subjects (smokers: 90; non-smokers: 94) were included considering their duration and frequency of smoking for conducting this study. The mean hearing thresholds of non-smoker subjects at 1, 4, 8 and 12 kHz frequencies were 5.63±2.10, 8.56±5.75, 21.06±11.06, 40.79±20.36 decibel (dB), respectively and that of the smokers were 7±3.8, 13.27±8.4, 30.66±12.50 and 56.88±21.58 dB, respectively. The hearing thresholds of the smokers at 4, 8 and 12 kHz frequencies were significantly (*p*<0.05) higher than those of the non-smokers, while no significant differences were observed at 1 kHz frequency. We also observed no significant difference in auditory thresholds among smoker subgroups based on smoking frequency. In contrast, subjects smoked for longer duration (>5 years) showed higher level of auditory threshold (62.16±19.87 dB) at 12 kHz frequency compared with that (41.52±19.21 dB) of the subjects smoked for 1-5 years and the difference in auditory thresholds was statistically significant (*p*<0.0002). In this study, the Brinkman Index (BI) of smokers was from 6 to 440 and the adjusted odds ratio showed a positive correlation between hearing loss and smoking when adjusted for age and body mass index (BMI). In addition, age, but not BMI, also played positive role on hearing impairment at all frequencies. Thus, these findings suggested that cigarette smoking affects hearing level at all the frequencies tested but most significantly at extra higher frequencies.

## Introduction

Hearing is one of the most important tools of social communication. Hearing is known to be deteriorated gradually during the ageing process [1]. In addition to ageing, a variety of environmental factors may also cause hearing impairment. Noise exposure has been proven to be the most important environmental factor for weakening hearing level [2]. In the United States 14% of workers work in an environment where the noise level exceeds 90 decibels (dB) according to a report of the National Institute for Occupational Safety and Health [3]. In Korea, workers with noise-induced hearing loss account for more than 90% of total workers with occupational diseases according to an earlier report [4]. In addition to the work-related exposure to noise, previous studies have shown that a modern lifestyle generally using MP3 player with an earphone is becoming a serious risk for hearing loss [5, 6]. On the other hand, smoking is also considered as one of the risk factors for hearing loss. A previous study has shown that light smoking classified by the Brinkman Index (BI; cigarettes/day multiplied by number of years) affects extra-high-frequency (12 kHz) auditory thresholds in young adults who have a lifestyle using portable MP3 player and drinking alcohol [7]. Other studies further examined the relationship between smoking and noise-induced hearing loss and showed that the incidence of noise-induced hearing loss is significantly higher in smokers [8–10] or that there is a synergistic effect between the two factors [11, 12]. Moreover, some studies showed a dose-response relationship between the amount of smoking and impairment of noise-induced hearing acuity [13]. Meanwhile, there is a previous study showing the absence of a significant correlation between the two factors [14]. Thus, the synergistic influence on hearing by smoking and noise-induced hearing loss remains controversial.

On the other hand, smoking cigarettes is considered as a potential risk factor for the most life-threatening chronic diseases including cancers (lung, throat, blood etc.), cardiovascular and respiratory diseases [15, 16]. In spite of these consequences, prevalence of smoking is very widespread around the world. In Bangladesh, more than 20% of populations are addicted to some form of smoking [17]. Prevalence of hearing impairment among South Asian population is also known to be quite high. However, the direct relationship between hearing impairment and smoking cigarettes remained mostly unfocused. Only few studies have been shown to cause hearing loss linking with cigarette smoking [7, 18–20]. Meanwhile, some studies failed to find any direct correlation between them [21, 22]. Most of the earlier studies have investigated the correlation of hearing impairment among subjects who have used MP3 player and have drunk alcohol. This study was therefore attempted to investigate whether cigarette smoking affects hearing among subjects in Bangladesh who maintain a lifestyle without using MP3 player and drinking alcohol. For this purpose, the study was conducted among 184 subjects of different ages, among them 90 subjects smoked cigarettes on daily basis. The audiometric measurement was taken at 1, 4, 8 and 12 kHz frequencies. The mean ± S.D values of auditory thresholds were measured followed by analysis of the correlation between hearing impairment and smoking cigarettes. Furthermore, the study was attempted to find out any association of hearing impairment with frequency and duration of cigarettes smoked.

## Methods

### Study Subjects

The study was conducted among 184 male subjects aged between 18–60 years who agreed in written to participate in audiometric testing. In this study, we did not include those subjects who had a habit of drinking alcohol and using portable music player with earphones. We also excluded those participants who had a previous history of ear diseases and suffered from illness at the time of survey. In addition, no subject was included in this study from other ethnic group or race. The body mass index (BMI) was calculated by using the formula: Weight in kg/(Height in metre^2^). All the experiments were undertaken considering the ethical issues and the study was approved by the Faculty of Biological Science, University of Dhaka (Ref. no. 5509/Bio.Sc). A survey was performed using a self-reporting questionnaire on smoking habit including duration of smoking, frequency of cigarette smoking/day, age, previous history of disease, weight and height of the participants.

### Measurement of Hearing Level

Measurement of hearing level at 1, 4, 8 and 12 kHz frequencies were performed in all the participating subjects. Audiometric examination was conducted in a sound-proof room using an iPod with earphones as described previously [23, 24]. Sound signals at 1, 4, 8 and 12 kHz frequencies were presented to each subject until the threshold of sound that the subjects were just able to perceive was identified. Hearing levels of all the subjects were measured by providing an initial 5 dB stimulus followed by stepwise increase in sound level by 5 dB. Examination of hearing was duplicated in each subject to confirm the repeatability of the values. The subjects were classified as having low/mild frequency hearing loss if the average of the pure-tone thresholds at 1 and 4 kHz frequencies were exceeded 20 dB. High frequency hearing loss was considered if the average of the pure-tone thresholds at 8 and 12 kHz frequencies were exceeded 40 dB. As described earlier, of the 184 subjects, 90 were smokers and the rest 94 were non-smokers control. The relationship between cigarette smoking and hearing level was evaluated using Brinkman index (BI) which was defined by the number of cigarettes smoked/day multiplied by the number of years [25]. According to BI, subjects were classified as a non-smoking group (BI = 0) and a smoking group (6 ≤ BI ≤ 440). Since the highest BI (e.g. 440) in this study was less than the BI (e.g. > 600) of defined heavy smokers in a previous report [26], we included all the smokers in smoking group rather than classifying them as heavy or light smoking groups.

### Data analysis

Data were statistically analyzed using SPSS program version 22 software (SPSS Inc., Chicago, USA). As the data did not show normal distribution, the difference between each group was analyzed using Pearson’s χ^2^ (chi-square) method. The descriptive statistics were also presented in the result. For each characteristics of the subjects, *p*-value and odds ratio were measured. For further confirmation, binary logistic regression analysis was performed to determine adjusted odds ratio and 95% confidence interval (CI). The regression analysis made use of the different predictor variables in the numerical form. Hearing level was taken as dependent variable, and smoking habit, age and BMI were considered as independent variables. The significance of the results was set at *p* < 0.05.

## Results

### Characteristics of the study population

Among 184 subjects analyzed, 90 (48.91%) were smokers and 94 (51.08%) were non-smokers. Of the smokers (n = 90), 49 (54.4%) were aged ≤40 years and the rest 41 (45.6%) were aged >40 years (Table 1). The mean age for the smokers was 39.07±11.6 years and that for the non-smokers was 36.34±12.2 years. BMI of the smokers and nonsmokers were 23.26 ± 3.3 and 23.78 ± 2.7 kg/m^2^, respectively. The subjects were further categorized as underweight, normal weight and overweight based on their BMI <18.5, 18.5–25 and >25 kg/m^2^, respectively. The number of subjects under normal weight of smokers and nonsmokers were 63 (70% of total smokers) and 62 (66% of total nonsmokers), respectively (Table 1), denoting the major portion of the subjects. The number of subjects under overweight category in both the smokers and nonsmokers were relatively low (smoker: 23, 25.6%; nonsmoker: 29, 30.9%). Least numbers of subjects were underweight in both of the smokers (4, 4.4%) and nonsmokers (3, 3.2%). *P*-values for comparing the mean age, age category, BMI and BMI category between smoker and nonsmoker are also shown in Table 1.

**Table 1:** Characteristics of the participants according to smoking status.

	Smoker	Nonsmoker	*p*-value
**Total No.**	90	94	
**Mean age (years)**	39.07±11.6	36.34±12.2	0.122
**Age category**			
≤40 years old: n (%)	49 (54.4%)	55 (58.5%)	0.07
>40 years old: n (%)	41 (45.6%)	39 (41.5%)	0.28
**BMI (kg/m** ^**2**^ **)**	23.26 ± 3.3	23.78 ± 2.7	0.241
**BMI category** ^**¶**^			
Under weight: n (%)	4 (4.4%)	3 (3.2%)	0.002
Normal weight: n (%)	63 (70%)	62 (66%)	0.003
Over weight: n (%)	23 (25.6)%	29 (30.9%)	0.007
**Duration of smoking**			
1–5 years: n	23		
>5 years: n	67		
**Frequency of smoking/day**			
1–10 cigarettes/day: n	35		
11–20 cigarettes/day: n	45		
>20 cigarettes/day: n	10		

^¶^The subjects were categorized underweight, normal weight and overweight when the BMI was found <18.5, 18.5–25 and >25 kg/m^2^, respectively.

### Cigarette smoking caused hearing impairment

**Fig 1:**
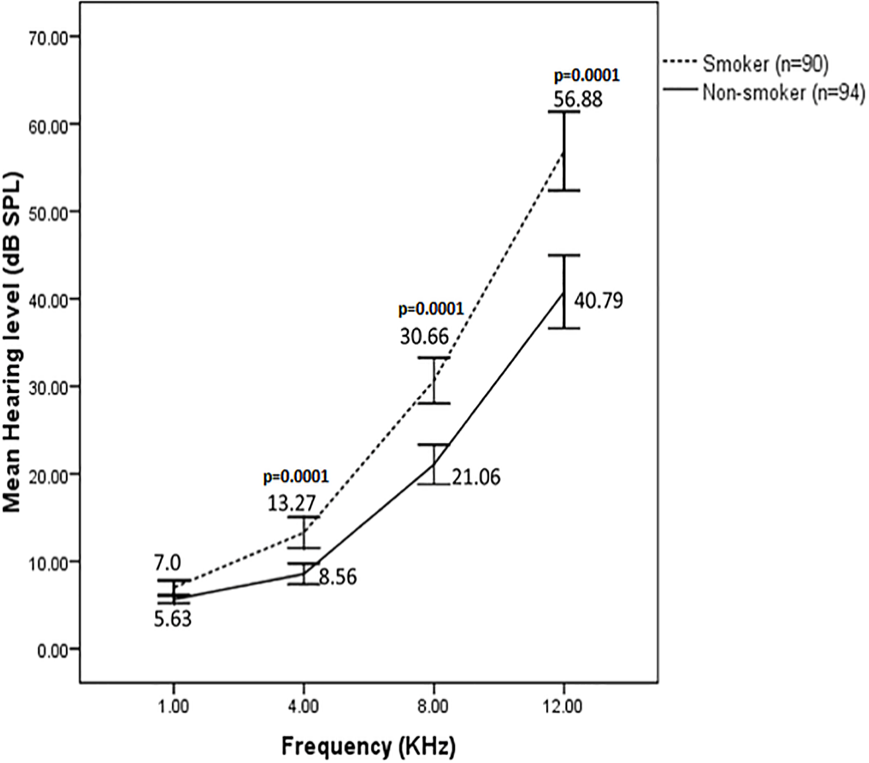
Effect of smoking on hearing level. Auditory thresholds (mean ± S.D) from 1 to 12 kHz frequencies in non-smoker ‘control’ (Brinkman index [BI = 0]; *n* = 94) and smokers (6 ≤ BI ≤ 440; *n* = 90) are shown. Smokers showed significantly (*p* = 0.0001) higher auditory thresholds than non-smokers at 4, 8 and 12 kHz frequencies.

In case of control (non-smoker) subjects (n = 94), the average auditory thresholds observed at 1, 4, 8 and 12 kHz frequencies were 5.63±2.10, 8.56±5.75, 21.06±11.06, 40.79±20.36 dB, respectively (Fig. 1). When the average auditory thresholds for smokers (n = 90) were measured at all of the frequencies above, we found the values as 7±3.8, 13.27±8.4, 30.66±12.50 and 56.88±21.58 dB, respectively. The hearing level is quantified relative to 'normal' hearing in dB, with higher values of dB indicating worse hearing. The average auditory thresholds for smokers were found significantly (*p* = 0.0001) higher than non-smokers at 8 and 12 kHz frequencies. The differences which might be attributed to smoking that smoking with BI≤440 caused impairment of hearing level at all the frequencies and that the hearing impairment was most evident at the higher frequency (12 kHz). Since the mean ± S.D of BI in this experiment was 129.25 ± 124.80, smoking group (n = 90) with an average BI of ~130 might cause hearing impairment at all frequencies compared to non-smokers.

### Correlation between age and smoking

The prevalence of hearing impairment was found higher among smokers of both age groups (≤40 and >40 years) compared with those of the nonsmokers at all the frequencies tested (Table 2). At 1, 4, 8 and 12 kHz frequencies, the percentage of the older smokers (>40 years) who experienced hearing loss was 14.6, 39.0, 68.3 and 92.7%, however, the values for those of the nonsmokers was 5.1, 10.3, 35.9 and 76.9%. In case of younger smokers (≤40 years), smoking also caused hearing loss compared with younger nonsmokers, however, the effect was relatively less than the older age groups at all the frequencies. The above results indicated a possible role of smoking in decreasing hearing level of the subjects regardless of their age. Moreover, the most profound effect of smoking on hearing for both age groups was observed at the extra high frequency (12 kHz). For both smokers and nonsmokers of older and younger groups, there was a significant difference (*p*<0.05) in hearing impairment at almost all of the higher frequencies. However, the difference was not found significant (*p* = 0.06) between smoker and nonsmoker older than 40 years at the extra higher frequency (12 kHz). As hearing loss was more profound for the older age group (nonsmoker: 76.9%, smoker: 92.7%) at that frequency, significant difference probably was not obtained.

**Table 2:** Association between smoking and age on hearing impairment.

Frequency	Age & smoking status	Subjects undergoing hearing impairment		*p*-value	Odds ratio (95% CI)
		No.	%		
**12 kHz**	**≤40 years**				
	Smoker (n = 49)	26	53.1	0.0003*	5.08
	Non-smoker (n = 55)	10	18.2		(2.09–12.33)
	**>40 years**				
	Smoker (n = 41)	38	92.7	0.06	3.80
	Non-smoker (n = 39)	30	76.9		(0.94–15.20)
**8 kHz**	**≤40 years**				
	Smoker (n = 49)	18	36.7	0.002*	5.80
	Non-smoker (n = 55)	5	9.1		(1.95–17.22)
	**>40 years**				
	Smoker (n = 41)	28	68.3	0.004*	3.84
	Non-smoker (n = 39)	14	35.9		(1.52–9.72)
**4kHz**	**≤40 years**				
	Smoker (n = 49)	10	20.4	0.03*	4.44
	Non-smoker (n = 55)	3	5.5		(1.14–17.23)
	**>40 years**				
	Smoker (n = 41)	16	39.0	0.005*	5.60
	Non-smoker (n = 39)	4	10.3		(1.67–18.77)
**1kHz**	**≤40 years**				
	Smoker (n = 49)	2	4.1	0.05	2.2
	Non-smoker (n = 55)	1	1.8		(0.20–26.15)
	**>40 years**				
	Smoker (n = 41)	6	14.6	0.17	3.1
	Non-smoker (n = 39)	2	5.1		(0.59–16.77)

*Statistically significant.

Abbreviation: CI: confidence interval; OR: odds ratio.

### Correlation between smoking frequency and hearing impairment

**Fig 2:**
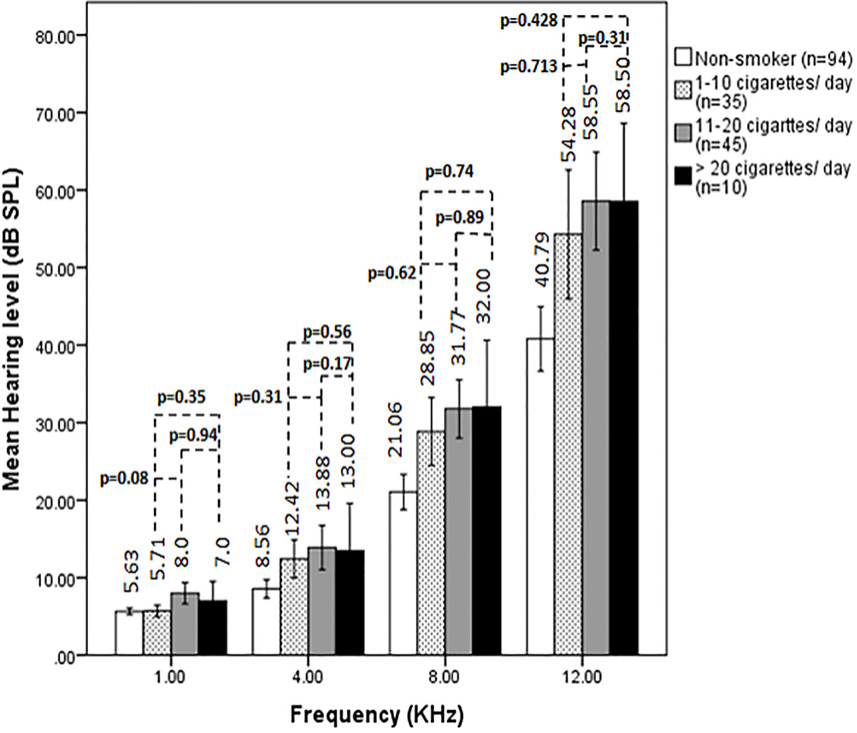
Effects of smoking frequency on hearing level. Auditory thresholds (mean± S.D) from 1 kHz to 12 kHz frequencies in non-smoker ‘control’ (n = 94) and smokers are shown. According to the frequency of smoking, smokers were divided into 3 subgroups as 1–10 cigarettes/day (n = 35), 11–20 cigarettes/day (n = 45) and >20 cigarettes/day (n = 10). The *p*-values for the difference in hearing thresholds among the smoker subgroups at all the frequencies tested were shown. *P*-values were calculated comparing between smoker subgroups 1–10 cigarettes/day and 11–20 cigarettes/day; 1–10 cigarettes/day and >20 cigarettes/day; and 11–20 cigarettes/day and >20 cigarettes/day.

We divided the smokers into 3 subgroups depending on their frequency of smoking per day (Table 1) to examine whether this frequency could influence hearing level. Although the average auditory thresholds were slightly increased with increasing smoking frequency, however; the difference in hearing thresholds among the smoker subgroups at all the frequencies (1, 4, 8 and 12 kHz) tested was not statistically significant (*p* > 0.05; Fig. 2). Though smoking generally affects hearing level, frequency of smoking (1 to >20 cigarette ranges/day) did not show significant additional impairment of hearing with increasing smoking amount.

### Duration of smoking impaired hearing level at high frequencies

**Fig 3:**
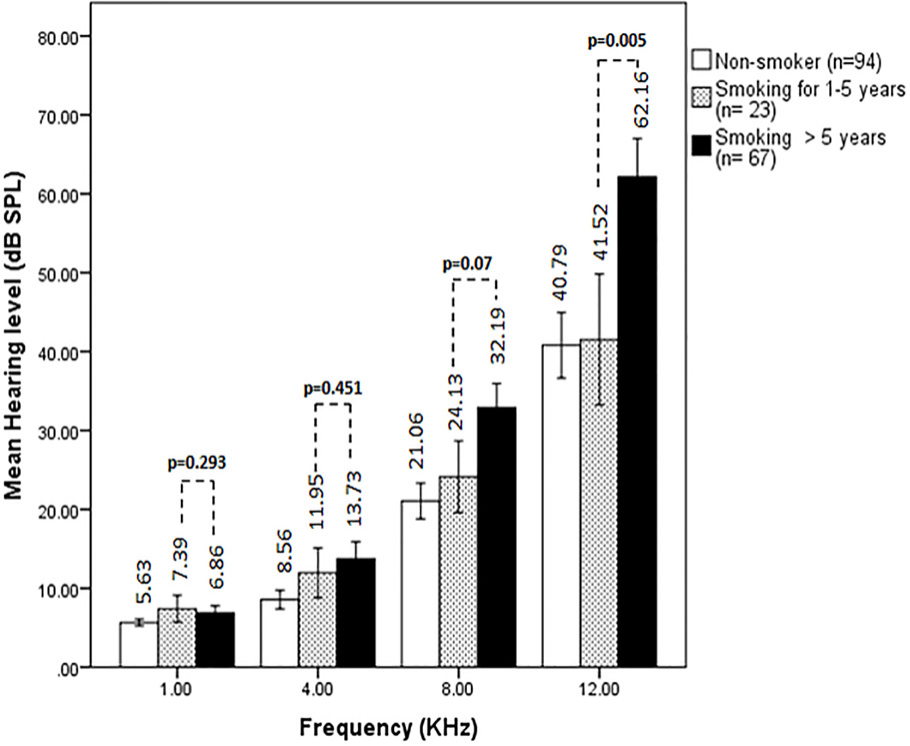
Effects of duration of smoking on hearing level. Auditory thresholds (mean± S.D) from 1 kHz to 12 kHz frequencies in non-smoker ‘control’ (n = 94) and smokers are shown. Based on duration of smoking, smokers were divided into 2 subgroups as smoked for 1–5 years (n = 23), and for more than 5 years (n = 67). The difference in auditory threshold between smokers smoked for 1–5 years and >5 years was statistically significant (*p* = 0.005) at 12 kHz frequency, however, the values were not found significant at all other frequencies (*p*>0.05).

The effect of smoking duration on hearing level was also examined. Smokers were divided into two subgroups (Table 1): smoking for 1–5 years and smoking for >5 years. Smoking duration was classified into 1–5 years and >5 years to denote smoking for shorter and longer period of time based on an earlier study that examined the influence of intensity and duration of smoking on recurrent aphthous stomatitis lesions [27]. The auditory thresholds at 8 and 12 kHz frequencies for smokers who smoked for 1–5 years (n = 23) were 24.13±10.15 and 41.52±19.21 dB, respectively, whereas the values for smokers who smoked for more than 5 years (n = 67) were 32.91±12.40 and 62.16±19.87 dB, respectively (Fig. 3). The difference in auditory threshold at 12 kHz frequency between smokers smoked for 1–5 years and more than 5 years was statistically significant (*p* = 0.005). However, at lower frequencies such as 1, 4 and 8 kHz, the results were not found significant (*p*-value 0.293, 0.451 and 0.07, respectively). As the auditory threshold (62.16±19.87 dB) for smokers who smoked for more than 5 years exceeded far from the normal value of 40 dB at 12 kHz extra high frequency, it might be concluded that smoking cigarettes for long time chronically affect hearing levels.

### Binary logistic regression analysis

Association of hearing loss with smoking was further examined by logistic regression. The hearing loss (>40 dB for 12 and 8 kHz frequencies and >20 dB for 4 and 1 kHz frequencies) was taken as dependent variable and smoking habit, age, BMI of the participants were considered as independent variables. The independent variables were categorized considering nonsmoker, age ≤40 years and BMI (normal weight) as reference group. Smoking and age were found to affect the hearing level more profoundly than the control groups after adjusting these factors (Table 3). The results suggested that controlling for differences in age and BMI of the participants, smoking increased the likelihood of hearing loss 4.90, 4.74, 5.04 and 2.85 times at 12, 8, 4 and 1 kHz frequencies respectively than non-smokers. Age also played positive role on hearing impairment at all frequencies. On the other hand, there were minor correlations of hearing loss with BMI (Table 3).

**Table 3:** Adjusted odds ratio for hearing level^¶^ at all frequencies.

Hearing level (n = 184) Adjusted OR (95% CI)	1kHz		4kHz		8kHz		12kHz	
	OR (95% CI)	*p-*value	OR (95% CI)	*p-*value	OR (95% CI)	*p-*value	OR (95% CI)	*p-*value
**Smoking habit**								
Non-smoker	1.0	0.14	1.0	0.0001*	1.0	0.0001*	1.0	0.0001*
Smoker	2.85		5.04		4.74		4.9	
	(0.7–11.3)		(2–12.5)		(2.3–9.6)		(2.3–10.5)	
**Age**								
≤40 years old	1.0	0.06	1.0	0.03*	1.0	0.0001*	1.0	0.0001*
>40 years old	3.71		2.38		4.14		12.75	
	(0.9–14.9)		(1.1–5.4)		(2.1–8.3)		(5.7–28.7)	
**BMI**								
Normal weight	1.0	0.99	1.0	0.78	1.0	0.86	1.0	0.30
Underweight	0.0		0.73		0.84		0.66	
			(0.7–7.0)		(0.13–5.2)		(0.1–4.0)	
Overweight	0.75	0.18	0.86	0.74	1.38	0.41	1.55	0.65
	(0.2–3.0)		(0.35–2.1)		(0.64–2.9)		(0.7–3.6)	

Abbreviation: CI: confidence interval; OR: odds ratio.

^¶^Adjusted for smoking, age and BMI.

*Statistically significant.

## Discussion

This study showed that smoking cigarettes significantly increased the risk of hearing impairment especially at higher frequencies. Among the smokers, 35% found to be smoked 1–10, 45% smoked 11–20 and 10% smoked more than 20 cigarettes daily. As compared with the non-smoker group, the smoker group was found at higher risk of developing hearing loss due to showing higher hearing thresholds at various frequencies tested. According to our results, the smokers with higher hearing thresholds at 4, 8 and 12 kHz might be more vulnerable for developing hearing loss due to showing significant difference in hearing levels in those frequencies. Our study correlates with some previous reports showing smoking mediated hearing loss at extra-high frequencies [7, 21, 28].

The association between smoking or alcohol consumption/exposure to occupational noise/use of MP3 player and hearing loss have been shown in earlier studies [3–11]. Ohgami et al. [7] demonstrated the effects of life style (with smoking, drinking, noise exposure and sleeping time) on hearing level in young adults with narrow age range of 21–23 years. This study was so far the first attempted to determine the relationship of smoking alone with hearing impairment in Bangladeshi population with a wider age range of 18–60 years. Therefore, we mainly included those subjects in this study who maintained a lifestyle devoid of using music player and alcohol drinking to exclude a possible synergistic role of these factors with smoking. Apart from the earlier report that smoking, but not noise exposure or sleeping time, significantly affects hearing level at extra high frequency (12 kHz) in young adults [7], our results demonstrated that smoking affected the hearing level more profoundly in subjects of a wider age range at all higher frequencies (Table 3). The duration and frequency of smoking were also considered as risk factors for evaluating hearing loss at higher frequency. We found that the average auditory thresholds were slightly increased with increasing smoking amount but the difference in hearing thresholds among the smoker subgroups at all the frequencies tested was not statistically significant (*p*>0.05; Fig. 2). However, some earlier studies have demonstrated that there is a significant correlation between the severity of hearing loss and smoking amount [3, 10]. In addition, a dose-response relationship between the amount of smoking and the impairment of hearing acuity has been reported in the presence [13] or absence of exposure to noise [18].

Some previous studies have been carried out to distinguish the synergistic effect on hearing impairment between smoking and aging or other related factors [12, 19, 29]. In accordance with the results of these previous studies, the present study also showed subjects that smoke with aged >40 years had a greater risk of hearing impairment compared to nonsmokers. Although hearing loss was observed in older nonsmokers as a function of age, the combined effects of smoking and age on hearing in smokers were more intense. When differences in age and BMI of the subjects were controlled, logistic regression analysis showed several fold increase in hearing loss by smoking compared to nonsmokers (OR: 4.90, 4.74, 5.04 and 2.85 at 12, 8, 4 and 1 kHz frequencies). Although the present study showed minor correlation of hearing loss with BMI, however, it was reported that BMI correlated well with higher hearing threshold across the whole frequency range in a large population-based study [30].

There are some limitations associated with the present study. The study was conducted using a self-reporting questionnaire on smoking habit, age and previous disease history of the participants. Data obtained through self-reporting were adopted directly without testing their authenticity by any other means. This limitation was probably minimized by guaranteeing the participants that their answers would be kept confidential. Apart from their smoking habit, the subjects with an earlier history of diseases were excluded and therefore the influence of this important risk factor on hearing could not be analyzed due to incompleteness of the data. No female subjects were included in this study mainly because female smokers are relatively rare in urban areas of Bangladesh. For this reason, we could not compare the effect of smoking on hearing level between male and female smokers. Nonetheless, based on the findings of the current study these limitations do not prevent to conclude that smoking impaired hearing level on the subjects with a wider range of age group (18–60 years).

Although the exact mechanism by which smoking affects the auditory organ is still unclear, several studies pointed out some mechanisms including direct ototoxicity of nicotine, cochlear ischemia due to increased levels of carboxyhemoglobin, and smoking-mediated increased blood viscosity [31–33]. Therefore, the involvement of these mechanisms collectively or individually might cause the smokers more vulnerable to hearing loss as compared with non-smokers. Further studies are needed to determine the molecular mechanism of smoking-mediated hearing impairment.

## Supporting Information

S1 Fig Similar frequency distributions of sound output between pure tone audiometry (PTA) and iPod. Frequency distributions (means ± SD) of tone burst sound (1–12 kHz) output by (A) PTA system and (B) iPod are presented. Both devices used earphone-type headphone (Panasonic RP-HJE150). The PTA system consists of PR2.1 Enhanced Real Time Processor, PA5 Programmable Attenuator and HB7 Headphone driver (Tucker-Davis Technologies, Inc). Sound levels from an earphone in a soundproof room were measured by a noise level meter (Type 6224 with an FFT analyzer, ACO CO., LTD, Japan) for 30 seconds and calculated as average of three repeated measurements. Background levels measured in a soundproof room without sound-generating devices were subtracted from sound levels from the earphone. Whole sound levels of (A) PTA and (B) iPod measured by the noise level meter without FFT analyzing software were almost the same (70 dB SPL).
(TIF)

S2 Fig Comparable hearing measurements between PTA and iPod. Hearing levels (1–12 kHz) of eight subjects (21 years old) measured by PTA (closed square) and iPod (open diamond) are presented.
(TIF)
