# Supplementary figures and images for: Cigarette Smoking Causes Hearing Impairment among Bangladeshi Population
Source: PLoS One. 2015 Mar 17;10(3):e0118960. doi: 10.1371/journal.pone.0118960 (PMC4363488; doi:10.1371/journal.pone.0118960)

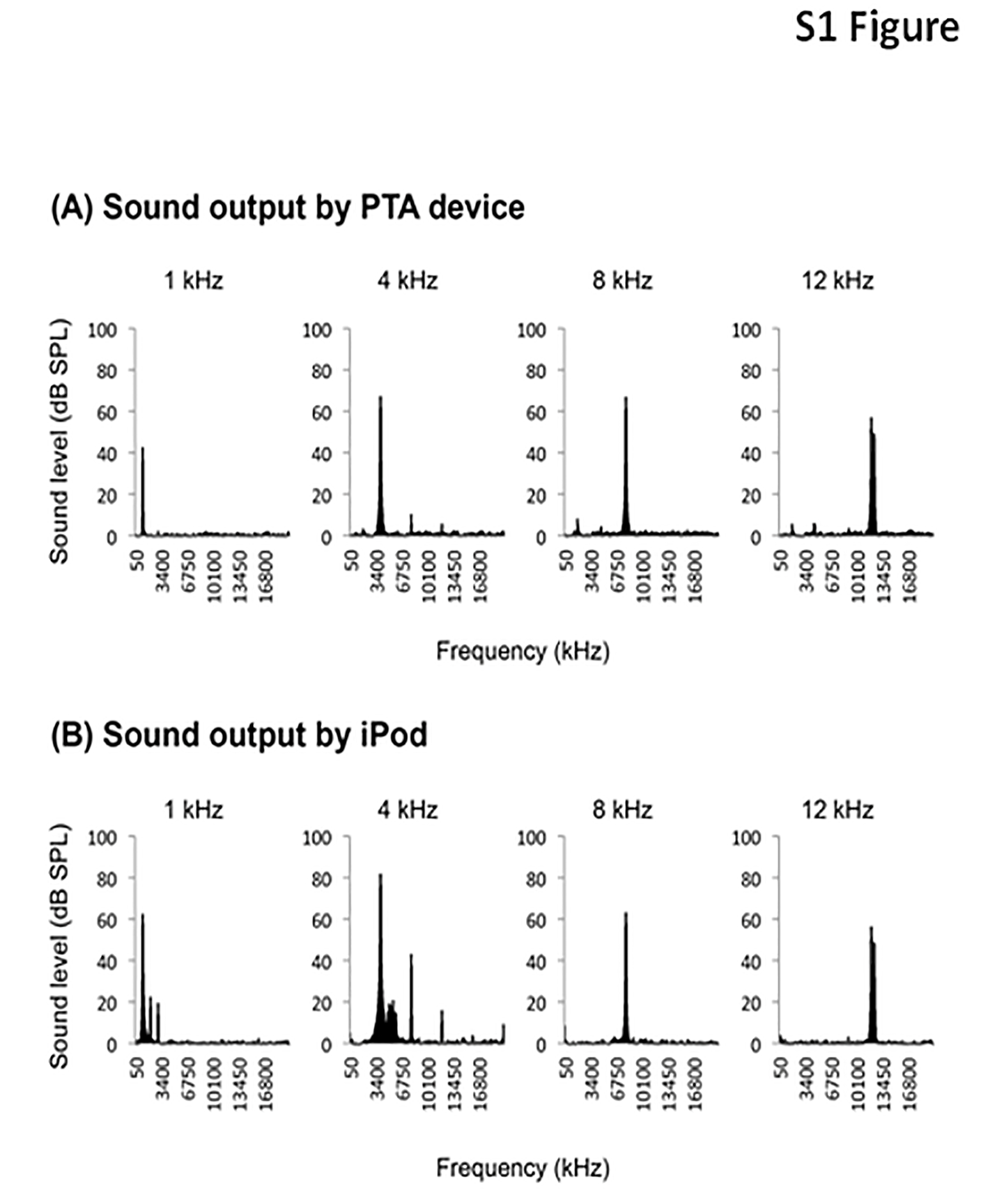

Supplement: S1 Fig — Frequency distributions (means ± SD) of tone burst sound (1–12 kHz) output by (A) PTA system and (B) iPod are presented. Both devices used earphone-type headphone (Panasonic RP-HJE150). The PTA system consists of PR2.1 Enhanced Real Time Processor, PA5 Programmable Attenuator and HB7 Headphone driver (Tucker-Davis Technologies, Inc). Sound levels from an earphone in a soundproof room were measured by a noise level meter (Type 6224 with an FFT analyzer, ACO CO., LTD, Japan) for 30 seconds and calculated as average of three repeated measurements. Background levels measured in a soundproof room without sound-generating devices were subtracted from sound levels from the earphone. Whole sound levels of (A) PTA and (B) iPod measured by the noise level meter without FFT analyzing software were almost the same (70 dB SPL). (TIF) [file pone.0118960.s001.tif]

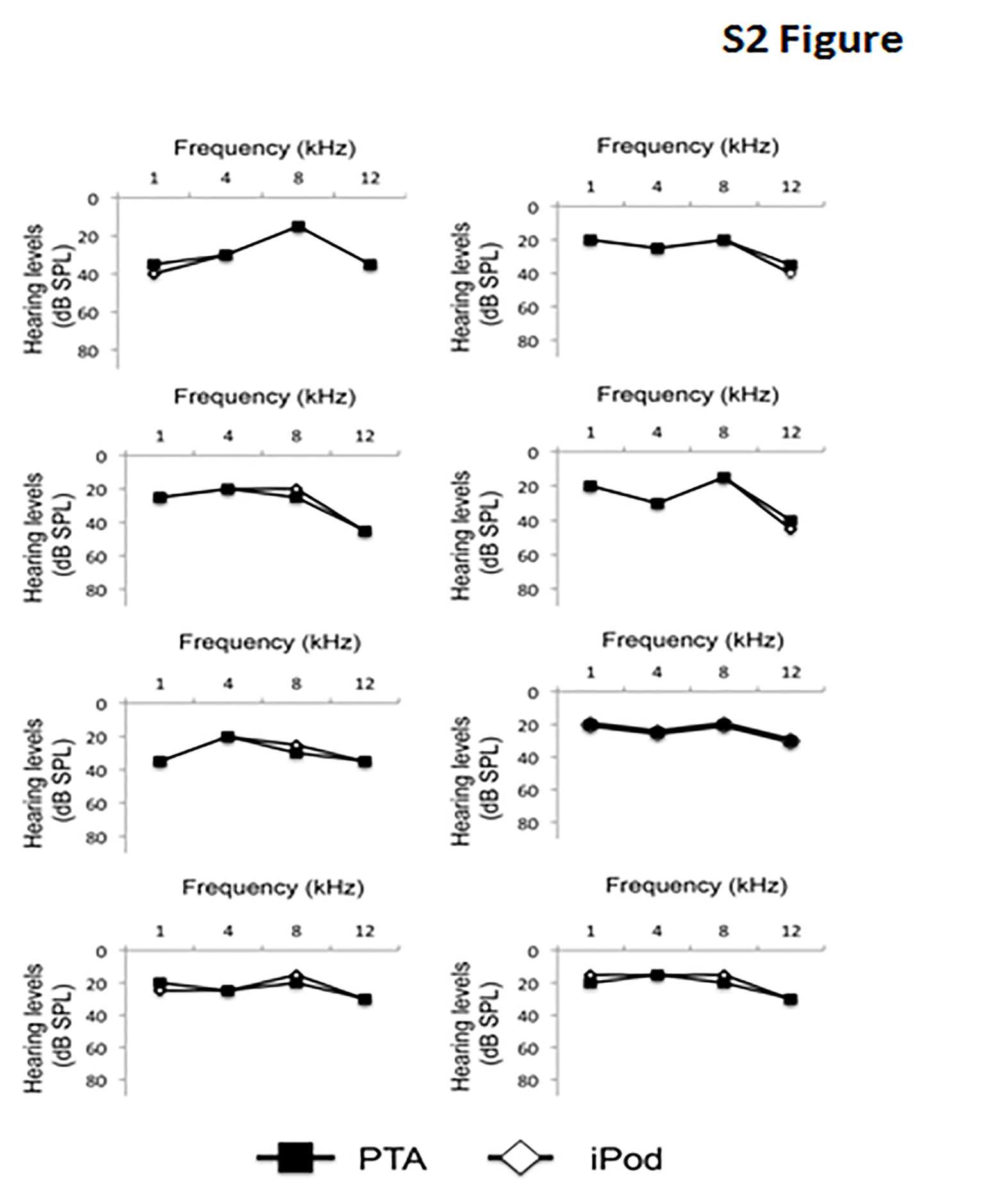

Supplement: S2 Fig — Hearing levels (1–12 kHz) of eight subjects (21 years old) measured by PTA (closed square) and iPod (open diamond) are presented. (TIF) [file pone.0118960.s002.tif]
